# Uncontrolled hypertension among tobacco-users: women of prime childbearing age at risk in India

**DOI:** 10.1186/s12905-021-01280-x

**Published:** 2021-04-09

**Authors:** Biplab K. Datta, Muhammad J. Husain

**Affiliations:** 1 Global Noncommunicable Diseases Branch, Division of Global Health Protection, Center for Global Health, Centers for Disease Control and Prevention, 1600 Clifton Road, Atlanta, GA 30329 USA; 2 Present Address: Institute of Public and Preventive Health, Augusta University, Augusta, GA USA

**Keywords:** Hypertension, Tobacco, Childbearing age, India

## Abstract

**Background:**

Uncontrolled hypertension and tobacco use are two major public health issues that have implications for reproductive outcomes. This paper examines the association between tobacco-use status and uncontrolled hypertension among prime childbearing age (20–35) women in India.

**Methods:**

We used the India National Family Health Survey (NFHS-4) 2015–2016 to obtain data on hypertension status and tobacco use for 356,853 women aged 20–35. We estimated multivariate logistic regressions to obtain the adjusted odds ratio for tobacco users in favor of having uncontrolled hypertension. We examined the adjusted odds at different wealth index quintiles, at different educational attainment, and at different level of nutritional status measured by body mass index.

**Results:**

We found that the odds of having uncontrolled hypertension for the tobacco user women in India was 1.1 (95% CI: 1.01–1.19) times that of tobacco non-users at prime childbearing age. The odds were higher for tobacco-users at the poorest quintile (1.27, 95% CI: 1.14–1.42) and with no education (1.22, 95% CI: 1.10–1.34). The odds were also higher for tobacco-users who were overweight (1.88, 95% CI: 1.57–2.29) or obese (2.82, 95% CI: 1.88–4.24).

**Conclusions:**

Our findings highlight the disproportionate dual risk of uncontrolled hypertension and tobacco use among lower-income women of prime childbearing age, identifying an opportunity for coordinated tobacco control and hypertension prevention initiatives to ensure better health of reproductive-age women in India.

## Background

Hypertension or elevated blood pressure is the most significant risk factor for almost all cardiovascular diseases (CVD) [1], the number one cause of death worldwide [2]. Uncontrolled hypertension is associated with an increased risk of CVD-specific mortality [3]. In addition to the risk of CVD, hypertension in women of childbearing age may complicate pregnancy and affect childbirth outcomes [4]. Tobacco use, both smoking and smokeless, is a risk factor for elevated blood pressure [5–7]. Tobacco use is also associated with increased risk of infertility, certain birth defects and adverse birth outcomes [8]. Both uncontrolled hypertension and tobacco use, thus have implications for childbearing-age women’s health and pregnancy outcomes.

The literature on tobacco use among childbearing-age women and hypertension in childbearing-age women separately examines the prevalence and determinants of each condition. Studies explore socioeconomic patterns of tobacco consumption [9], tobacco use during pregnancy [10], types of tobacco use (smoking- or smokeless-tobacco) and geographic correlates [11, 12], educational and wealth inequalities [13] associated with tobacco use, and the association of tobacco use with child mortality [14], food insecurity [15], and intimate partner violence [16]. Studies on hypertension investigate hypertension awareness and control among reproductive-age women [17], urban-rural [18] and cross-country [19] differences in prevalence, risk factors associated with undiagnosed hypertension [20], and the association of hypertensive condition with obesity [21], socioeconomic gradients [22], wealth and education-based inequalities [23], and household environment [24]. Though certain aspects of tobacco use and hypertension in reproductive-age women were studied individually, there is a gap in terms of assessing the dual risk of tobacco use and uncontrolled hypertension.

One such entwined aspect previously not explored in literature is whether women of prime childbearing age (20–35), who consume tobacco products, have an added risk of being hypertensive and not having it under control. Childbearing during adolescent age (15–19) has certain risks and complications [25]. Giving birth at advanced maternal age (over 35) is also associated with adverse pregnancy outcomes [26]. On the other hand, childbearing during age 20–35 is comparatively safe and regarded optimal for pregnancy outcomes [27]. In the absence of maternal age gradient being a prominent risk factor, uncontrolled hypertension among tobacco user women has implications for reproductive health outcomes in this age group.

We explore this issue using data from India, the second-most populous country in the world, where female tobacco use, hypertension, and child and maternal health are critical public health issues. India has a very large tobacco-user female population with approximately 65 million (14.2%) women aged 15 and older consuming smoking and/or smokeless tobacco [28]. A recent study estimates that 95 million (20.0%) women aged 18 and older in India are hypertensive [29]. Among the reproductive-age females, nearly half are unaware of their hypertension status, and only one quarter receive treatment for lowering blood pressure or have their blood pressure under control [17]. Hypertensive disorders in pregnancy, which are linked to pre-existing hypertension [30], are among the leading causes of maternal death in India [31]. India also has the highest number of stillbirths globally [32], for which hypertension [33] and tobacco use [34] are critical risk factors. The adverse health outcomes of uncontrolled hypertension among childbearing-age women can be further aggravated by tobacco use, and vice versa.

Given the consequences of tobacco use and uncontrolled hypertension concerning maternal and child health, this study aims to assess the population level joint risk of tobacco use and uncontrolled hypertension in women at their prime age of childbearing. More than 75% of the births in India occur at mothers’ prime childbearing age [35], and, therefore, it is worthwhile to understand any population level risks that may impact pregnancy outcomes. Knowledge about whether a tobacco user female has an added risk of uncontrolled hypertension, thus have important implications for effectively managing maternal and child healthcare, particularly in a low resource setting. To the best of our knowledge, this is the first study to assess the joint risk of tobacco use and uncontrolled hypertension in women of prime childbearing age in a developing country. The findings of this analysis can inform policies for integrated and coordinated hypertension management and tobacco control strategies targeted toward childbearing-age women in developing countries.

## Methods

### Data

We used data from the National Family Health Survey (NFHS-4) 2015–2016 of India, a nationally representative survey that reports indicators of tobacco use and hypertension in reproductive-age women [35]. For our analysis, we obtained the tobacco use and hypertension status of 356,853 women aged 20–35. NFHS-4 collected self-reported information on respondents’ current use of smoked tobacco products (e.g., cigarette, bidi, cigar, pipe, hookah) and smokeless tobacco products (e.g., paan masala or gutkha, khaini, paan with tobacco, chewing tobacco, snuff, and other). The survey also provides blood pressure measures of reproductive-age women. Respondents’ systolic blood pressure (SBP) and diastolic blood pressure (DBP) were measured three times during a single visit with at least five minutes interval between each measure. Survey protocols of the NFHS-4, which is part of the USAID’s Demographic and Health Surveys (DHS) program, were reviewed and approved by the ICF Institutional Review Board (IRB). Details of the ethical review are available at: https://dhsprogram.com/What-We-Do/Protecting-the-Privacy-of-DHS-Survey-Respondents.cfm.

### Measurement

The NFHS-4 questionnaire asks whether the respondent currently smokes or uses tobacco in any other form. An individual was identified as a tobacco user if she reported current (at the time of the survey) consumption of one or more of the tobacco products mentioned above. For hypertensive condition, individuals with SBP $\ge 140 \quad mmHg$ or DBP $\ge 90 \quad mmHg$ were identified to have uncontrolled hypertension. We applied the following algorithm that replicates the estimates in the NFHS-4 final report to account for SBP and DBP for each woman: we took the “first measure” if available and the second and third measures were missing (1.1% of the sample); the “second measure” if available and the third measure was missing (1.9% of the sample); the “third measure” if available and the second measure was missing (0.2% of the sample); and the average of the “second and third measures” if both were available (94.3% of the sample) [35].

### Statistical analysis

Using the NHFS-4 data, we estimated differences in uncontrolled hypertension prevalence between tobacco user and non-user females. We examined these differences by wealth index quintiles for the full sample and for urban and rural sub-samples. These differences, however, do not account for the sociodemographic characteristics of the respondents. To obtain adjusted measures of differences in uncontrolled hypertension status between the tobacco-user and non-user groups, we estimated a multivariate logistic model. Our outcome variable is a binary variable indicating whether the respondent had uncontrolled hypertension or not. The tobacco use variable is denoted by another binary variable indicating any tobacco (smoking or smokeless) product consumption by the respondent at the time of the survey.

We controlled for a rich set of covariates including age group (20–24 (base category), 25–29, or 30+), respondent’s nutritional status measured by body mass index (BMI) categories (underweight: $<18.5$ kg/m$^2$, normal: 18.5–24.9 kg/m$^2$ (base category), overweight: 25.0–29.9 kg/m$^2$, or obese: $\ge$ 30.0 kg/m$^2$), wealth index quintiles (base category: 1st *quintile*), educational attainment (no education (base category), primary, secondary, or higher), current marital status (never married (base category), currently married, or widowed/divorced/separated), and urban or rural residence. We also controlled for state fixed effects to control for state-level variations in women’s health-related issues in India. The multivariate logistic model was separately estimated for the full sample, and for urban and rural sub-samples using complex survey weights. The adjusted odds ratios in favor of having uncontrolled hypertension were reported.

Next, we examined the adjusted odds ratios for the tobacco use variable in favor of having uncontrolled hypertension at different wealth index quintiles, at different levels of educational attainment, and at different levels of nutritional status (measured by BMI). For these analyses, we interacted the tobacco-use indicator with respective indicator variables and estimated separate multivariate logistic specifications for each indicator (i.e., wealth index, educational attainment, and BMI group). The exponentiated value of the coefficient of the interaction term is the adjusted odds ratio for the tobacco use variable at the respective category of the respective indicator.

The set of sociodemographic controls excludes the *j*th indicator for respective specifications. For example, it does not include wealth index quintiles while the model was estimated to examine the odds ratios of tobacco use at different wealth index quintiles. Lastly, we estimated the multivariate logistic specification for mutually exclusive tobacco-use categories by replacing “any tobacco-use” indicator with three types of tobacco-use indicators—“smoking-only”, “smokeless-only”, and “dual-use”. All these specifications were estimated for the full sample and urban and rural sub-samples using complex survey weights.

## Results

### Descriptive analysis

In the NFHS-4, 5.4% of the prime childbearing-age women reported some kind of tobacco use (smoking and/or smokeless). More than 80% of the tobacco users consumed smokeless tobacco only, nearly 16% consumed smoking tobacco only, and around 2% consumed both smoking and smokeless tobacco (dual-use). Tobacco use among prime childbearing-age women in India was more prevalent in the rural areas than in urban areas, and in the poorest quintiles than in wealthier quintiles (Table 1). Nearly half of the tobacco users did not have any formal education. Tobacco use was more prevalent in the older (age 30+) age group, and almost 90% of the tobacco users were married at the time of the survey.

**Table 1:** Descriptive statistics by tobacco user and non-user

	Non-user	Tobacco-user	All
	*Share* (%)		
Urban	34.97	22.41	34.29
	(34.52, 35.41)	(21.29, 23.54)	(33.85, 34.73)
*Age group*			
20–24	34.63	19.79	33.82
	(34.38, 34.88)	(19.07, 20.52)	(33.58, 34.07)
25–29	31.75	30.65	31.69
	(31.52, 31.99)	(29.75, 31.56)	(31.46, 31.92)
30+	33.62	49.55	34.48
	(33.36, 33.87)	(48.53, 50.57)	(34.24, 34.73)
*Nutritional status*			
Normal (BMI 18.5–24.9)	60.37	57.48	60.21
	(60.10, 60.65)	(56.58, 58.38)	(59.95, 60.48)
Underweight (BMI < 18.5)	20.86	31.52	21.43
	(20.63, 21.08)	(30.67, 32.37)	(21.21, 21.65)
Overweight (BMI 25.0–29.9)	14.63	8.88	14.32
	(14.42, 14.84)	(8.32, 9.44)	(14.12, 14.53)
Obese (BMI $\ge$ 30.0)	4.14	2.12	4.03
	(4.01, 4.27)	(1.80, 2.44)	(3.90, 4.16)
*Wealth index quintile*			
1st Quintile	16.38	40.08	17.67
	(16.13, 16.64)	(39.07, 41.09)	(17.41, 17.93)
2nd Quintile	18.83	28.19	19.34
	(18.58, 19.09)	(27.36, 29.02)	(19.09, 19.59)
3rd Quintile	20.93	17.59	20.75
	(20.66, 21.21)	(16.83, 18.34)	(20.49, 21.02)
4th Quintile	22.18	10.03	21.53
	(21.87, 22.50)	(9.32, 10.74)	(21.21, 21.84)
5th Quintile	21.66	4.12	20.71
	(21.26, 22.07)	(3.61, 4.62)	(20.32, 21.10)
*Education*			
No education	21.99	48.27	23.42
	(21.73, 22.26)	(47.17, 49.37)	(23.15, 23.70)
Primary	12.48	20.76	12.93
	(12.29, 12.67)	(20.01, 21.51)	(12.74, 13.11)
Secondary	46.79	28.81	45.82
	(46.45, 47.14)	(27.85, 29.77)	(45.48, 46.15)
Higher	18.73	2.16	17.83
	(18.42, 19.05)	(1.83, 2.48)	(17.53, 18.14)
*Marital status*			
Never in union	15.06	6.48	14.60
	(14.84, 15.28)	(6.00, 6.96)	(14.39, 14.81)
Married	82.58	88.71	82.91
	(82.35, 82.81)	(88.09, 89.34)	(82.69, 83.14)
Widowed, divorced/separated	2.36	4.81	2.49
	(2.27, 2.44)	(4.40, 5.21)	(2.41, 2.57)
Observations	323,937	32,916	356,853

95% confidence intervals are in parenthesis

Estimates were obtained using complex survey weights

### Bivariate analysis

**Fig. 1:**
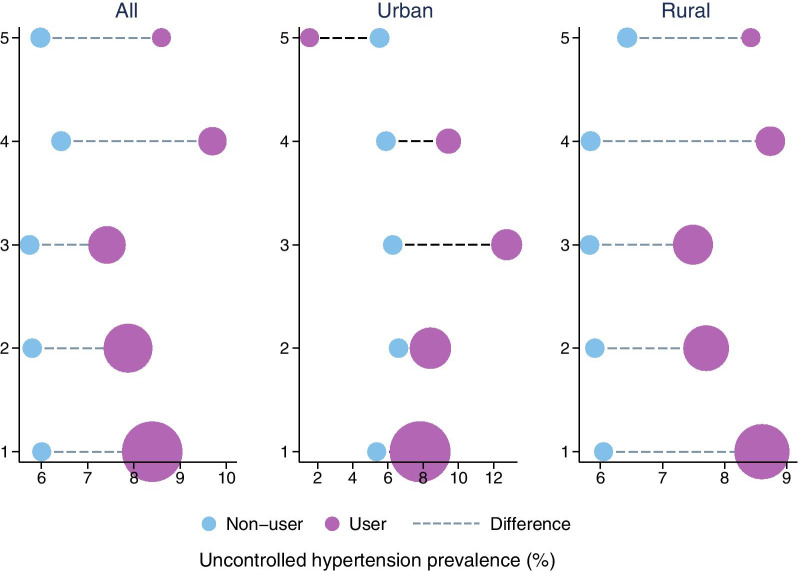
Difference in hypertension prevalence between tobacco-user and non-user by wealth index quintiles. Estimates were obtained using complex survey weights. The size of the bubbles reflects share of tobacco-users or non-users across wealth index quintiles. The shares of tobacco-users across quintiles add to one. The shares of non-users across quintiles add to one

From the blood pressure measures, it was estimated that around 6% of the age 20–35 females in India had uncontrolled hypertension. However, among tobacco users, this rate was as high as 8%. Figure 1 shows the differences in uncontrolled hypertension prevalence between tobacco users and non-users across wealth index quintiles. The difference in uncontrolled hypertension prevalence ranges from 1.7 to 3.3 percentage points across quintiles. The gap exists at every wealth quintile in both urban and rural areas except the urban top quintile where the prevalence is higher among tobacco non-users. This may be due to very low level of tobacco consumption at the urban top quintile (0.70%) and proportionately fewer hypertensive cases in this small group.

### Multivariate analysis

Table 2 presents the adjusted odds ratios in favor of having uncontrolled hypertension. The odds of having uncontrolled hypertension for tobacco users was 1.10 times that of tobacco non-users. The odds were slightly lower in the rural areas (1.08) and slightly higher (1.15) though not statistically significant ($p=0.202$) in the urban areas. These outcomes were adjusted for potential confounders such as age, wealth, and education. Among other covariates, the odds were higher for the older age individuals compared to age 20–24, and for overweight and obese individuals compared to individuals of normal BMI.

**Table 2:** Adjusted odds ratios

	All	Urban	Rural
Tobacco user	1.097**	1.151	1.079*
	(1.014, 1.186)	(0.928, 1.428)	(0.998, 1.166)
Age group			
25–29	1.574***	1.752***	1.500***
	(1.479, 1.675)	(1.526, 2.011)	(1.406, 1.601)
30+	2.576***	2.953***	2.422***
	(2.431, 2.730)	(2.594, 3.362)	(2.277, 2.576)
*Nutritional status*			
Underweight (BMI < 18.5)	0.700***	0.658***	0.710***
	(0.662, 0.740)	(0.562, 0.770)	(0.669, 0.752)
Overweight (BMI $25.0-29.9$)	2.170***	2.100***	2.215***
	(2.053, 2.295)	(1.892, 2.330)	(2.086, 2.352)
Obese (BMI $\ge$ 30.0)	3.261***	3.066***	3.443***
	(2.993, 3.553)	(2.694, 3.489)	(3.075, 3.855)
*Wealth index quintile*			
2nd Quintile	0.885***	1.173**	0.901***
	(0.832, 0.941)	(1.029, 1.338)	(0.839, 0.968)
3rd Quintile	0.866***	1.122*	0.853***
	(0.810, 0.927)	(0.984, 1.279)	(0.792, 0.920)
4th Quintile	0.955	1.063	0.869***
	(0.886, 1.030)	(0.915, 1.234)	(0.801, 0.943)
5th Quintile	0.902**	1.056	0.901**
	(0.825, 0.986)	(0.899, 1.239)	(0.827, 0.981)
*Education*			
Primary	0.967	0.905	0.987
	(0.907, 1.031)	(0.772, 1.060)	(0.923, 1.056)
Secondary	0.827***	0.865**	0.799***
	(0.782, 0.875)	(0.763, 0.981)	(0.751, 0.850)
Higher	0.663***	0.661***	0.677***
	(0.606, 0.726)	(0.562, 0.778)	(0.613, 0.748)
*Marital status*			
Married	0.848***	0.826**	0.846***
	(0.784, 0.918)	(0.713, 0.956)	(0.778, 0.920)
Widowed, divorced/separated	0.949	0.897	0.961
	(0.826, 1.089)	(0.699, 1.150)	(0.818, 1.128)
Urban	0.915***		
	(0.863, 0.971)		
Constant	0.049***	0.035***	0.052***
	(0.037, 0.065)	(0.021, 0.058)	(0.038, 0.071)
Observations	356,853	103,690	253,116

95% confidence intervals are in parenthesis

Estimates were obtained using complex survey weights

All models control for state fixed effects

***$$p<0.01$$, **$$p<0.05$$, *$$p<0.1$$

**Fig. 2:**
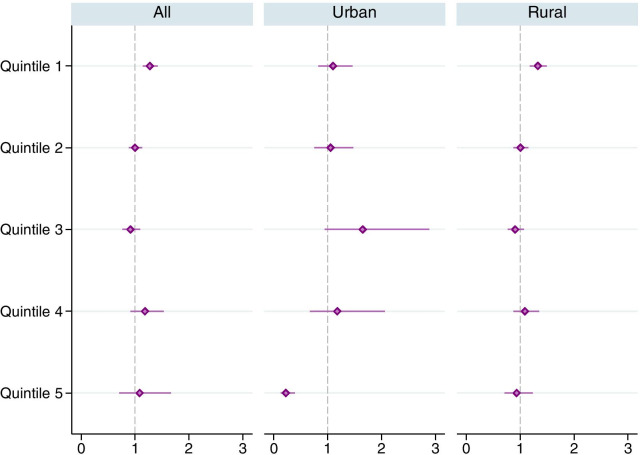
Adjusted odds ratios for any tobacco use in favor of uncontrolled hypertension, by wealth index quintiles. The horizontal lines around the markers represents 95% confidence intervals. Estimates were obtained using complex survey weights. Regressions controlled for age group, nutritional status (BMI), education, marital status, urban/rural residence and state fixed effects

Across the wealth index quintiles, the odds of having uncontrolled hypertension for tobacco users were the highest (1.27) at the poorest (1st) quintile (Fig. 2). The odds were also highest (1.33) at the poorest (1st) quintile in the rural areas. Among urban quintiles the odds were the highest (1.65) at the middle (3rd) quintile and the lowest at the top (5th) quintile. These outcomes were adjusted for potential confounders such as age and education.

**Fig. 3:**
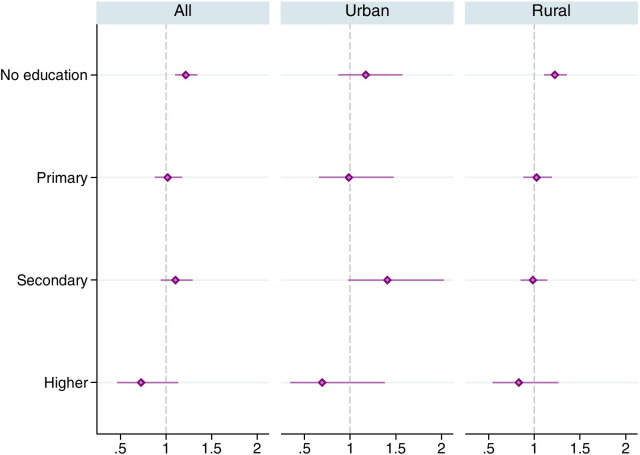
Adjusted odds ratios for any tobacco use in favor of uncontrolled hypertension, by educational attainment. The horizontal lines around the markers represents 95% confidence intervals. Estimates were obtained using complex survey weights. Regressions controlled for age group, wealth index quintiles, nutritional status (BMI), marital status, urban/rural residence and state fixed effects

The odds of having uncontrolled hypertension for tobacco users were the highest (1.22) for individuals having no formal education (Fig. 3). However, for the urban sub-sample, the odds were the highest (1.41) for individuals with secondary level of education. The odds for the higher educated group were lower and statistically insignificant for both urban and rural sub-groups. These outcomes were adjusted for potential confounders such as age and wealth.

**Fig. 4:**
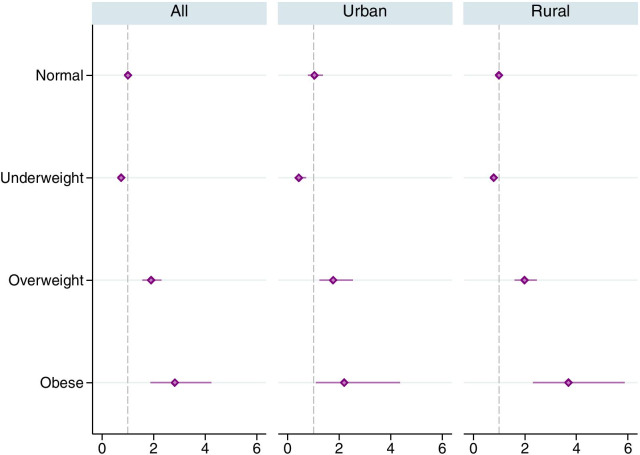
Adjusted odds ratios for any tobacco use in favor of uncontrolled hypertension, by nutritional status (BMI group). The horizontal lines around the markers represents 95% confidence intervals. Estimates were obtained using complex survey weights. Regressions controlled for age group, wealth index quintiles, education, marital status, urban/rural residence and state fixed effects

The odds were the highest (2.82) for obese tobacco users followed by overweight tobacco users (1.90) and the lowest (0.74) for the underweight tobacco users (Fig. 4). The findings were consistent in both urban and rural sub-samples. These outcomes were adjusted for probable confounders such as age, wealth, and education.

**Fig. 5:**
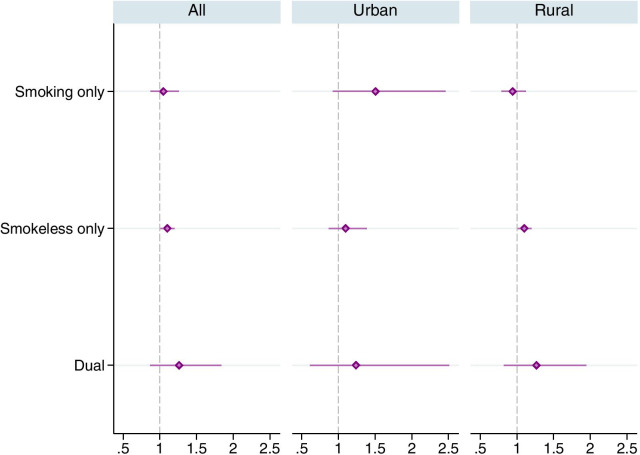
Adjusted odds ratios for mutually exclusive types of tobacco use in favor of uncontrolled hypertension. The horizontal lines around the markers represents 95% confidence intervals. Estimates were obtained using complex survey weights. Regressions controlled for age group, nutritional status (BMI), wealth index quintiles, education, marital status, urban/rural residence and state fixed effects

Finally the odds for users of smokeless-only were 1.10 times of that of the tobacco non-users in the full sample as well as in the rural sub-sample (Fig. 5). The odds for smokeless-only use were not statistically significant ($p=0.437$) in the urban sub-sample. The odds for dual-use were higher across all samples but not statistically significant (*p* value ranges from 0.222 to 0.283). The odds for smoking-only were, however, higher (1.51, $p=0.103$) in the urban sub-sample.

## Discussion

This paper investigated the association between two important public health issues—uncontrolled hypertension and tobacco use among prime childbearing-age women in India. We, however, do not study whether tobacco use causes hypertension. Rather we examine whether tobacco-user women in India at their prime age of childbearing have an additional risk of uncontrolled hypertension. We find that there is a statistically significant association between being a tobacco user and having uncontrolled hypertension among women aged 20–35. Our findings describe the dual problem of uncontrolled hypertension in prime childbearing age women in India who use tobacco products, demonstrating the need for greater access and utilization of hypertension care in this group.

Childbearing-age tobacco-user females in India predominantly consume smokeless tobacco products. The nicotine, sodium, and licorice contents of smokeless tobacco products can aggravate hypertensive conditions [36]. Prolonged use of smokeless tobacco products is also associated with an increased risk of fatal myocardial infarction and fatal stroke [37]. Despite the adverse health effects of smokeless tobacco use, implementation and enforcement of smokeless tobacco control policies in India remained challenging, mainly because of the fear of economic consequences of job losses among workers in the smokeless tobacco manufacturing sector [36]. Inadequate tobacco control measures to curb smokeless tobacco use may, therefore, contribute to the risk of cardiovascular diseases among childbearing-age women in India. In addition to the adverse reproductive outcomes associated with smokeless tobacco use during pregnancy, the disproportionately high prevalence of uncontrolled hypertension among tobacco users may further compound the risk of adverse pregnancy outcomes.

The greater odds of having uncontrolled hypertension for the tobacco user female at prime age of childbearing could have consequences for infant and maternal health in India, where neonatal mortality (24.9 per 1000 live births) and maternal mortality (130 per 100,000 live births) are serious health concerns [38, 39]. The association between tobacco use and uncontrolled hypertension represents an additional challenge to safe motherhood in India, motivating policy attention to improving maternal health and childbirth outcomes.

An important aspect of our findings is the relatively higher likelihood of uncontrolled hypertension among overweight and obese tobacco users than non-users. The risk of adverse pregnancy outcomes is higher among obese women [40] and may be further increased by tobacco use and uncontrolled hypertension condition. We also find that the likelihood of having uncontrolled hypertension was relatively higher for tobacco-user women at the lowest wealth index quintile and with no education, highlighting a source of health disparities in India. There are persistent socioeconomic inequalities in tobacco use prevalence in India, raising questions about the reach of the tobacco control measures to poor and uneducated populations [41]. The association between tobacco use and uncontrolled hypertension, therefore, could further worsen the high burden of adverse health conditions for the socioeconomically disadvantaged populations in India.

One limitation of this analysis is that blood pressure in NHFS-4 was measured during one occasion, which may result in an incorrect determination of hypertension status for some respondents. The data on tobacco use were self-reported, which could be subject to some measurement error. We also do not know if a respondent quit tobacco within weeks or months prior to the interview date. Due to the cross sectional nature of the data we could not offer any causal relationship between tobacco use and uncontrolled hypertension. We also did not analyze the relationship between exposure to secondhand smoking and uncontrolled hypertension, which may be studied in future research. However, despite these limitations we document an important association that has consequences for public health and requires policy attention of the public health practitioners.

## Conclusions

The disproportionately high level of uncontrolled hypertension among prime-childbearing-age women, who consume tobacco, highlights a dual public health risk that motivates health policy attention. The prime childbearing age of 20–35 is regarded as the safest for pregnancy outcome. We provide evidence that tobacco user women of this age group in India have an additional risk of uncontrolled hypertension, which may exacerbate the health risks of tobacco use concerning child and maternal health. Documentation of this dual risk lays out new avenues of research in public health practice that may lead to better management of maternal and child health issues. Our findings, in general, inform policies for coordinated tobacco control and hypertension prevention initiatives. Tobacco control interventions targeted toward socioeconomically disadvantageous women combined with improved access to hypertension treatment could support efforts to improve health outcomes in India.

## Data Availability

The datasets used and/or analyzed during the current study are available from the USAID’s Demographic and Health Surveys (DHS) Program. The DHS datasets are free to download and use upon registering at the DHS program website: https://dhsprogram.com/data/new-user-registration.cfm.

## Abbreviations

CVD: Cardiovascular disease
NFHS-4: India National Family Health Survey Round 4
DHS: Demographic and Health Surveys
SBP: Systolic blood pressure
DBP: Diastolic blood pressure
BMI: Body mass index
